# Validation of the Chinese SAD PERSONS Scale to predict repeated self-harm in emergency attendees in Taiwan

**DOI:** 10.1186/1471-244X-14-44

**Published:** 2014-02-17

**Authors:** Chia-Yi Wu, Hui-Chun Huang, Shu-I Wu, Fang-Ju Sun, Chiu-Ron Huang, Shen-Ing Liu

**Affiliations:** 1 Department of Nursing, College of Medicine, National Taiwan University, Taipei, Taiwan; 2 Department of Medical Research, Mackay Memorial Hospital, Taipei, Taiwan; 3 Mackay Junior College of Medicine, Nursing and Management, New Taipei City, Taiwan; 4 Department of Psychiatry, Mackay Memorial Hospital, Taipei, Taiwan; 5 Department of Audiology and Speech Language Pathology, Mackay Medical College, New Taipei City, Taiwan

**Keywords:** Psychometric properties, Repetition, Self-harm, Suicide risk assessment, Taiwan

## Abstract

**Background:**

Past and repeated self-harm are long-term risks to completed suicide. A brief rating scale to assess repetition risk of self-harm is important for high-risk identification and early interventions in suicide prevention. The study aimed to examine the validity of the Chinese SAD PERSONS Scale (CSPS) and to evaluate its feasibility in clinical settings.

**Methods:**

One hundred and forty-seven patients with self-harm were recruited from the Emergency Department and assessed at baseline and the sixth month. The controls, 284 people without self-harm from the Family Medicine Department in the same hospital were recruited and assessed concurrently. The psychometric properties of the CSPS were examined using baseline and follow-up measurements that assessed a variety of suicide risk factors. Clinical feasibility and applicability of the CSPS were further evaluated by a group of general nurses who used case vignette approach in CSPS risk assessment in clinical settings. An open-ended question inquiring their opinions of scale adaptation to hospital inpatient assessment for suicide risks were also analyzed using content analysis.

**Results:**

The CSPS was significantly correlated with other scales measuring depression, hopelessness and suicide ideation. A cut-off point of the scale was at 4/5 in predicting 6-month self-harm repetition with the sensitivity and specificity being 65.4% and 58.1%, respectively. Based on the areas under the Receiver Operating Characteristic curves, the predictive validity of the scale showed a better performance than the other scales. Fifty-four nurses, evaluating the scale using case vignette found it a useful tool to raise the awareness of suicide risk and a considerable tool to be adopted into nursing care.

**Conclusions:**

The Chinese SAD PERSONS Scale is a brief instrument with acceptable psychometric properties for self-harm prediction. However, cautions should be paid to level of therapeutic relationships during assessment, staff workload and adequate training for wider clinical applications.

## Background

Suicide can cause significant years of life lost and bring impacts to service providers and the society as a whole [1]. The challenge of high suicide numbers but relatively few research in Asian countries have called for more attention in prevention strategies in Asia [2]. In this context, predicting suicidal behaviour for appropriate interventions has become an emerging issue for the clinicians [3,4]. Given the limited evidence on suicide risk assessment in predicting risk levels [5-7], more research are needed to investigate the validity of risk screening tools.

Past and repeated self-harm or suicide attempts were long-term risks to completed suicide [8-10]. Recognizing repetition risks of suicide by non-mental health professionals was an important variable that could improve patient outcome [11]. Such risk identification task can be performed through the nurses and motivated general health workers [12,13]. A simple scale and adequate risk assessment skills for first-line physicians and nurses would provide basic objective indicators of risk level [14] and raise awareness of the clinicians to offer professional support and attention to facilitate help-seeking behaviour among people with self-harm [15]. Though there was limited evidence on the accuracy of screening tools [7], risk screening under guidance of an effective and efficient tool appeared to be a critical and promising method for high-risk identification, follow-ups and further interventions [16,17].

Few established scales of suicide risk assessment were developed and studied [18], with some assessing indirect risks such as suicide ideation [19], depression or other general health indicaters [20]. Though depression scales such as the Patient Health Questionnaire provided acceptable diagnostic properties [21], its predictive capability may not be equal to that of direct risk assessment given reliable responses acquired. Supporting evidence for suicide scale validation was still limited [22]; even more limited in the Chinese populations. Moreover, few studies compared the differences among scales that directly assess suicide-related risks such as suicide intention (e.g. the Pierce Suicide Intention Scale or Beck Suicide Intention Scale), hopelessness (e.g. the Beck Hopelessness Scale) or a relatively comprehensive suicide risk assessment (e.g. the SAD PERSONS Scale) that was originally developed to assess the need to be hospitalized among emergency attendees with self-harm. The aim of the study was to demonstrate the psychometric properties and an adequate cut-off point of the Chinese version of the SAD PERSONS Scale (CSPS) to predict 6-month self-harm repetition and its applicability in general medical settings.

## Methods

### Study settings

The study was performed in northern Taiwan where the latest suicide rate was reported at 14.7 per 100,000 in 2011 [23]. The healthcare and medical context of the study has been previously reported [24]. A general assessment approach of suicide risk factors for consecutive patients with self-harm in the emergency department of Mackay Memorial Hospital (MMH) has been adopted since 2005. The researchers at the MMH suicide prevention center used the Modified SAD PERSONS scale [25] for suicide risk screening and provided service referral and case management interventions. In order to test the applicability and reliability of the scale, the first author further collected a group of general nurses’ opinions towards the use of the CSPS in another general hospital, the National Taiwan University Hospital (NTUH), which situated in the same area as another study hospital. In order to assure our study quality, the authors have established standardized procedure for assistant training and made sure of the consistency in data collection to avoid information bias. Moreover, the researchers who performed CSPS assessment were blinded of the baseline CSPS scores in order to derive reliable results in 6-month predictive performance of the CSPS assessment.

### Study subjects and procedures

The ethical approval was acquired from the two study hospitals respectively (reference numbers: MMH-I-S-202 and 201107036RB) before participants recruitment. The patients and nurses were interviewed by two trained research assistants and the first author respectively at the two study hospitals after they provided written consent forms. All participants with self-harm (the cases) admitted to the emergency department and patients without self-harm (the controls) from the family medicine department of the MMH provided informed consent. Self-harm was defined as infliction of a harmful act upon oneself in any form, self-poisoning or self-injury, with a non-fatal outcome [26]. Patients with self-harm who admitted to the emergency department were assessed by research assistants irrespective of the presence of suicide idea and based on self-inflicted harming behavior rather than diagnosis; those who denied self-harm were judged if the acts exceeded the tolerance of a person and caused harm. The control group was defined as those with general medical diagnoses without prior self-harm acts and was adopted based on the rationale of establishing the differentiation validity of the CSPS. The overall data quality was ensured by the corresponding author who supervised the entire study procedures in the suicide prevention center. Patients who were unable to complete their first interviews during hospital contact were further arranged an interview during outpatient visit within one week of their admission or at their homes. On the other hand, we randomly sampled 54 general nurses to join interactive group discussions using clustered randomization drawn from Medical, Surgical, Emergency/Intensive Care sectors in the NTUH. The main methodology description of the group intervention was specified elsewhere (under submission) and briefly introduced below. Each group was consisted of 5–10 nurses who joined two similar group sessions discussing case scenario using the CSPS. The first session included instruction of suicide epidemiology and discussion of how and what to assess the case’s suicide risk factors, and the second session strengthened the concepts of risk assessment using the same approach. After each group session, a satisfaction survey was carried out to evaluate the nurses’ opinions of the group intervention and the applicability and feasibility of using the CSPS. The CSPS applicability and reliability were then analyzed.

### The scale applicability and reliability

Using case vignette approach invented by the research team that describes medical inpatients with potential suicide risk factors (see an example in the Appendix), the first author discussed these factors and how they could be assessed by the general nurses in three interactive discussion groups. The pilot results showed that the scale of CSPS was generally accepted by the nurses who acknowledged its application in daily nursing care [15]. In this study we attempted to present the nurses’ evaluation towards the use of the underlying CSPS risk assessment in the post-intervention satisfaction survey and to analyze the test-retest reliability of their ratings at baseline and 3-week reassessment. The reliability was tested given that the correct answers of each CSPS item rating and the nurses’ rating performances were blinded to them.

### Measurements

The patients were interviewed at baseline by the CSPS and other psychometric scales measuring depression, suicide ideation and hopelessness as shown below. We followed up all the patients using all the scales at the sixth month of baseline interviews and evaluated self-harm repetition and the change in scores. Besides, we collected the nursing participants’ evaluations towards the CSPS using a response sheet in the satisfaction survey.

#### The Chinese version of SAD PERSONS scale (CSPS)

This 10-item mnemonic scale was designed for non-mental health professional use in assessing the overall suicide risk level among emergency attendees. The sensitivity and specificity were 94% and 71% respectively in the original study [25]. The cut-off of over 6 points of score was found to identify the need for hospitalization among emergency patients who self-harm [27,28]. Upon permission acquired from the scale inventor, we performed scale translation into Chinese version and the back translation, which reflected the same meanings of the contents in the original scale based on the researchers’ consensus; we then created inquiries for each item for the research assistants to draw responses from the patients. These inquiries were based on the mnemonic contents and were developed into semi-structural format. Each item was scored 2, 1 or 0 with the sum of 14 (see Additional file 1); higher scores indicate higher suicide risk level. Next, we performed a pilot testing of the applicability of CSPS in clinical setting by general nurses and found it a feasible and brief tool for quick assessment towards the inpatients’ suicide risk [15]. The rationale of selecting this scale for validation was that it could inform suicide risk level in self-harm patients who seek emergency care [25] or predict repetition of self-poisoning [29]. It has acceptable face validity and provides an opportunity for initial psychosocial assessment in clinical settings for people with suicide risks.

#### Pierce suicide intent scale (PSIS)

The PSIS encompasses 12 items that assess four aspects of suicide intent: self-harm context (item 1–4), suicide warning signs (item 5–6), subjective perceptions (item 7–10), and medical seriousness (item 11–12). It is notable that only those with actual self-harm acts can be rated using this scale rather than those with suicide ideation. Each item was scored 2 or 3 with a sum of 25 points, with higher scores indicating higher level of intent [30]. The internal consistency tested in this study was 0.81.

#### Hamilton rating scale for depression (HAMD)

This clinician-rated questionnaire was used to measure severity of depressive symptoms. We used the 24-item Chinese version for validation with other psychometric scales in this study [31]. Each question has 3–5 possible answers, with higher scores indicating higher levels of depression. The trained clinician must choose the possible responses to each question through interviews and by observation. It has been demonstrated to have a good inter-rater and internal reliability as well as satisfying concurrent validity [31]. The internal consistency tested in this study was 0.86.

#### Beck scale for suicide ideation (BSSI)

This 19-item instrument was developed to measure a person’s severity of suicide ideation and his/her plans and wishes to commit suicide [32]. All items were administered via semi-structural interviews by trained clinicians. Each item was rated 0–2, deriving a total score of 0–38 with higher score indicating severer conditions. The BSSI was shown to have good internal consistency with the Cronbach’s alpha values over 0.90s in the original study [32] as well as in ours (Cronbach’s α = 0.93).

#### Beck hopelessness scale (BHS)

This 20-item self-report inventory was designed to measure three major aspects of hopelessness: feelings about the future, loss of motivation, and expectations [33]. Each item requires a true (score 1) or false (score 0) response and is scored for indications of pessimism or denial of optimism. The total score ranged between 0–20, with lower scores indicating higher levels of hopelessness. It had good internal reliability and fair concurrent validity with the CSPS in this study. The internal consistency of this scale was found to be 0.82 (positive items) and 0.84 (negative items) in this study.

#### The satisfaction survey sheet

In a response sheet, we used a 5-point rating scale to measure questions evaluating the nurses’ opinions towards the applicability of the CSPS risk assessment in clinical settings. The items in the sheet included, for example, the depth and width of the CSPS and how they perceived the feasibility and applicability of adopting such scale in their units in the hospital. Moreover, we further designed an open-ended question asking about their suggestions towards scale applicability and opinions of each CSPS item (Additional file 1).

### Data analysis

The sociodemographic and background information regarding self-harm risk were analyzed using the SPSS 16.0. Among the cases with self-harm, we checked the concurrent validity of the CSPS with PSIS, HAMD, BSSI, BHS and also confirmed the predictive validity with the number of self-harm acts during 6-month follow-up period. We evaluated the associations of continuous variables by Pearson’s correlation and compared the means or percentages between groups of self-harm and non-self-harm using Independent *T*-test or Chi-squared test. In order to determine the optimal cut-off points for the CSPS in predicting the 6-month self-harm repetition, we compared predictive performance of the CSPS with other psychometric scales using areas under the Receiver Operating Characteristic (ROC) curves via the R Project for Statistical Computing. Furthermore, we presented sensitivity, specificity, positive predictive values and negative predictive values to decide a suitable cut-off score for future self-harm risk detection. Besides, two main parts of the nursing data were analyzed, i.e. the test-retest reliability and the nurses’ opinions from the satisfaction survey. The first author calculated mean difference of the ratings at two time points, three weeks apart, to evaluate the differences in rating performance and the stability of simulated nursing assessment by applying the CSPS in the case vignette approach. We also calculated the mean scores of the 5-point ratings (0–5 points) of the nurses’ opinions towards scale feasibility and applicability in clinical settings. Higher scores indicates that the nurses perceived the scale as more useful and suitable to be adopted in nursing assessment. Moreover, the open-ended question about their responses of scale adaptation to hospital inpatient assessment for suicide risks and suggestions were analyzed using content analysis.

## Results

The patient participants were consisted of 147 people with self-harm from the Emergency Room at baseline and the 6^th^ month and collected 284 patients without self-harm from the Family Medicine Department in the same hospital. The response rate at 6-month follow-up was 74.8%. Table 1 showed sample characteristics. In this female predominating (72.1%) sample mean-aged 35.6 (standard deviation 13.9) years, the 6-month repetition rate was 21.8% (regardless of self-harm types).

**Table 1:** Demographic information and self-harm risks of people with or without self-harm

	**Case (n = 147)**	**Control (n = 284)**	***X***^***2***^/t *	**p-values**
Age, mean ± S.D.	35.6 ± 13.9	38.3 ± 15.3	1.81	0.07
Gender (%)				
Female	106 (72.1)	184 (64.8)	2.36	0.13
Male	41 (27.9)	100 (35.2)		
Marital status (%)			27.83	<0.001
Single	56 (38.1)	152 (53.5)		
Married/cohabitated	61 (41.5)	115 (40.5)		
Divorced/separated/widowed	30 (20.4)	17 (6.0)		
Education years, mean ± S.D.	11.1 ± 3.1	13.7 ± 3.5	7.41	<0.001
Religious belief (%)			1.11	0.78
None	61 (41.5)	121 (42.6)		
Christianity/Catholic	15 (10.2)	39 (13.7)		
Buddhism/Daoism	70 (43.0)	122 (43.0)		
Other	1 (0.7)	2 (0.7)		
Self-harm methods (%)		-		
Prescribed medication	96 (65.3)			
Non-prescribed medication	12 (8.2)			
Self-cutting	37 (25.2)			
With alcohol consumption	26 (17.7)			
Charcoal burning	11 (7.5)			
Chemical substance poisons	5 (3.4)			
Drowning/suffocation	2 (1.4)			
The CSPS scores/items	4.54 ± 1.94	1.60 ± 1.11	−20.10	<0.001
High-risk group^	72 (49.0)	7 (2.5)	197.99	<0.001
1: Sex	41 (27.9)	100 (35.2)	2.36	0.13
2: Age	45 (30.6)	102 (35.9)	1.21	0.27
3: Depression/service use	96 (65.3)	19 (6.7)	170.24	<0.001
4: Prior self-harm/suicide	110 (74.8)	28 (9.9)	187.85	<0.001
5: Ethanol or drug use	50 (34.0)	4 (1.4)	93.96	<0.001
6: Rational thinking loss	5 (3.4)	1 (0.4)	6.56	0.01
7: Separated/widowed/divorced	100 (68.0)	169 (59.5)	3.00	0.08
8: Organized act	15 (10.2)	0 (0)	30.03	<0.001
9: No social supports	31 (21.1)	4 (1.4)	50.28	<0.001
10: Stated future intent	30 (20.4)	2 (0.7)	54.71	<0.001

*Chi-squared test for categorical variables or independent *t*-test for continuous variables were performed to derive the statistics of *X*^*2*^ or t respectively.

^The cut-off value used to define higher risk group was over 5 points; see Additional file 1 for the contents of each item.

*Abbreviations:* S.D.: standard deviation; CSPS: The Chinese version of SAD PERSONS Scale.

### Reliability of the psychometric measures in this study

Table 2 shows the results of internal consistency of main scales used for assessing the participants’ mental health status or suicide risks. The findings indicated that all the scales had good reliability performance with the Cronbach’s alpha values ranged between 0.82-0.93. The CSPS was not examined using internal consistency because each item was independent and derived from a variety of suicide risk factors.

**Table 2:** Internal consistency of the psychometric scales at baseline (n = 147)

	**Cronbach’s α**	**Item numbers**	**Case numbers**
PSIS	0.81	12	146
HAMD-24	0.86	24	147
BSSI	0.93	19	142
BHS (positive items)	0.82	8	142
BHS (negative items)	0.84	12	142

*Abbreviations:* CSPS (Chinese version of the SAD PERSONS Scale); PSIS (Pierce Suicide Intent Scale); HAMD (Hamilton Rating Scale for Depression); BSSI (Beck Suicide Scale Ideation); BHS (Beck Hopelessness Scale).

### Validity and reliability of the CSPS

Among people with self-harm, the total score of the CSPS were significantly correlated with all other psychosomatic scale scores at baseline, with a few scales moderately associated with the CSPS at 6-month follow-up assessment (i.e. all the r values were at 0.40s) (Table 3). The CSPS had low association with the scores of the PSIS (r = 0.19). In terms of the difference between the correlation coefficients of baseline and 6-month values, we found that both the scores of 6-month HAMD and BHS had stronger associations with the CSPS, while the predictive validity of BSSI remained stable (i.e. the *r* value was 0.42 at baseline and 0.41 at 6-month follow-up). On the other hand, the 3-week test-retest reliability revealed that the two ratings by 54 general nurses were significantly different (r = 0.12, p = 0.38), indicating its stability and reliability in repeated measurements.

**Table 3:** Concurrent validity of the Chinese SAD PERSONS with other psychometric scales

**Scale**^**^**^	**Baseline (n**^**⋄**^**)**	**6-month follow-up (n)**
PSIS	0.19^*^ (145)	-
HAMD	0.31^***^(144)	0.41^**^(108)
BSSI	0.42^***^(140)	0.41^*^ (109)
BHS	0.21^***^(140)	0.41^**^(105)

^*^P < 0.05 ^**^p < 0.01^ ***^p < 0.001; ^⋄^: case number; ^^^Pearson’s correlation coefficients.

### The receiver operating characteristic (ROC) curves

Using the ROC curves to estimate the optimal cut-off point of the CSPS, Table 4 indicated relatively acceptable psychometric values at cut-off 4/5 to predict repetition of self-harm within a 6-month follow-up (i.e. score over 5 and above refers to the high-risk group). With this cut-off value, clinicians could have 65.4% sensitivity and 58.1% specificity to predict 6-month repetition by any self-harm method. From clinical perspectives and based on the research team’s consensus, the positive and negative predictive values were also relatively acceptable at this cut-off point.

**Table 4:** Performance of predictive values (%) of the SAD PERSONS 10-item score towards 6-month self-harm acts (n = 147)

**Cut-offs**	**Sensitivity**	**Specificity**	**PPV**	**NPV**
Score 5/6	46.2	79.1	50.0	72.4
Score 4/5	65.4	58.1	50.0	72.4
Score 3/4	84.6	33.7	56.5	68.4

*Abbreviations:* PPV (Positive predictive value); NPV Negative predictive value).

**Figure 1:**
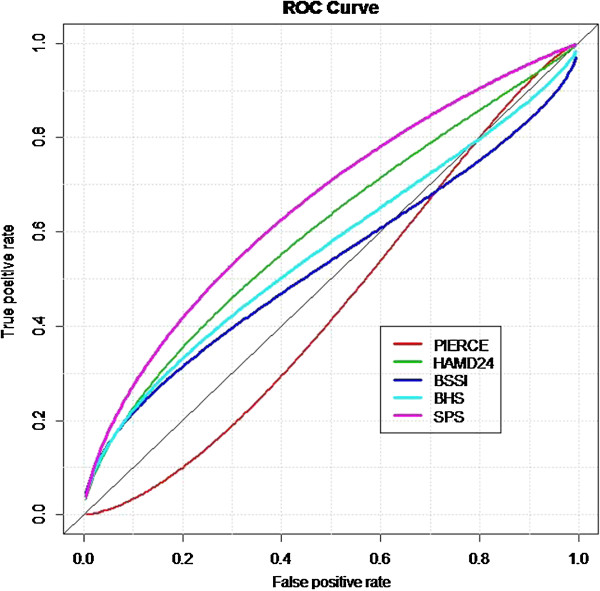
**The ROC curves of different scales predicting 6-month self-harm repetition.** Abbreviations: PIERCE (Pierce Suicide Intent Scale); HAMD24 (Hamilton Rating Scale for Depression); BSSI (Beck Suicide Scale Ideation); BHS (Beck Hopelessness Scale); SPS (The Chinese version of the SAD PERSONS Scale).

Moreover, Table 5 showed the performance of the CSPS scores in predicting 6-month self-harm act when comparing to the other four psychometric scales. Judged by the areas under the curves (AUCs) derived from the ROC method, we found that the CSPS had relatively better performance in prediction (AUC = 0.66, p = 0.013, 95% Confidence intervals = 0.54-0.78), followed by the BSSI, HAMD, BHS, and PSIS. We presented the visualized ROC curves in Figure 1. It appeared that the CSPS was a relatively considerable scale to be used for self-harm repetition prediction in this sample.

**Table 5:** Area under the curves (AUCs) to predict the 6-month self-harm act

			**95% Confidence interval**	
**Scale**	AUCs*	**p-value**	**Lower bound**	**Upper bound**
CSPS (n = 112)	0.66	0.02	0.53	0.79
PSIS (n = 112)	0.49	0.88	0.36	0.61
HAMD (n = 112)	0.56	0.40	0.42	0.70
BSSI (n = 109)	0.59	0.18	0.45	0.74
BHS (n = 109)	0.55	0.47	0.42	0.70

*Receiver Operating Characteristics method was used to generate the results.

*Abbreviations:* CSPS (Chinese version of the SAD PERSONS Scale); PSIS (Pierce Suicide Intent Scale); HAMD (Hamilton Rating Scale for Depression); BSSI (Beck Suicide Scale Ideation); BHS (Beck Hopelessness Scale).

### The nurses’ evaluations of clinical applicability of the CSPS

The main results of the nursing intervention were reported elsewhere. In this study we simply presented the findings according to the current study aims. The mean scores of their perceptions of feasibility and applicability were both 4.5 points. They perceived the CSPS as a brief framework and useful guide in nursing assessment for hospital inpatients. They revealed that it is a reminder of suicide risk factors based on which they could provide timely and effective referral after assessment. Many participants recognized its feasibility across different wards and applicability in daily nursing assessment given heavy nursing workloads. Under the challenge of patient characteristics and illness features in various wards, they acknowledged the need to enhance suicide risk awareness of the patients via using this short scale and also disclosed the need for adequate training (see Additional file 1).

## Discussion

The study provided evidence in identifying an optimal cut-off value of the CSPS to predict self-harm repetition with an acceptable concurrent and predictive validity. The qualitative findings also highlighted its applicability in general medical settings by non-mental health professionals. The scale was relatively brief and valid as well as adequate for suicide risk assessment in the clinical sample. Given its low internal consistency derived from different components of risk factors, the ten-item scale appeared to predict a higher risk for self-harm repetition. At cut-off of 5 point and above, the CSPS could reasonably classify a relatively high-risk group for future self-harm.

The findings of the CSPS validation were limited to comparability due to rarity of such studies [7,34]. In a Canadian study, the CSPS was not found to accurately predict future suicide attempts owing to a low sensitivity (40%) and low positive predictive value (PPV) (7.4%) [22]. In applying the 2 point cutoff approach to predict repetition as used in the above study, our results similarly indicated that the CSPS had good classification performance (sensitivity 56.5%, PPV 87.4%) and supported the predictive value of the scale. But we failed to proof its predictive performance by logistic regression analysis suggested by the above study in using separate CSPS items or total score alone to predict 6-month repetition, as we found only the item of Depression could predict future self-harm risk rather than many other items (data shown on request). Although using the CSPS alone might result in considerable false-positives, personnel workload, and extra resources allocated in staff training, it was evident that the scale was comparatively an appropriate option for suicide risk prediction than other scales tested in our study. Besides, the relatively low predictive ability of the PSIS shown in Figure 1 worth attention; it was possibly related to item contents which rated the severity of suicidality from one single self-harm episode that mostly reflects cross-sectional severity rather than a person’s consequent suicide risk level. Whereas in comparing to other scales with the CSPS, it also performed significantly better than depression, hopelessness, or suicide ideation measurements in self-harm prediction. Therefore it would be beneficial to build up staff awareness and train the first-line clinicians to use the CSPS for suicide risk assessment. This implication was similar to that of the others which emphasizing the importance of nesting risk screening in routine care by general nurses in general medical settings [35,36]. But according to nurses’ opinions, specific training may be needed on how to establish therapeutic relationship and get reliable responses for Item 4, 8, 10 in the CSPS.

The study was to the best of our knowledge the first that applied a relatively comprehensive method to validate the CSPS in a Chinese population and provided evidence-based data for its validation. We believe that the concerns for low PPV of the CSPS might be significantly improved if risk assessment is combined with another short scale for suicide risk evaluation such as depression. The strategy of augmenting risk prediction ability by using two brief and valid tools concurrently to screen for suicide risk level was also suggested by other study [37]. For example, one may include the CSPS with another brief depression scale such as the BSRS [19,38], the PHQ-2 [39] or other tool specifically for a certain age group such as the elderly (e.g. the Geriatric Depression Scale) to increase precision in predicting the risk level. The CSPS might provide a wider clinical usage in the general medical context for suicide risk identification rather than focusing on people with psychiatric illness, given that the latter group of people had different set of risk factors compared to the general population [34].

The study was among the few that discussed validation of the SAD PERSONS scale and presented both concurrent and predictive validity for relatively objective conclusions. The strengths included that it explored feasibility and applicability of clinical use in general hospitals and involved service providers’ (i.e. the nurses’) opinions which complement the study results. Our findings were salient in its mixed data sources with rigorous quality control performed across two major general hospitals. However, the results should be interpreted under several limitations. Firstly, it could be a concern that we tested the scale validity in the emergency department at one hospital but evaluated its applicability based on the nurses’ opinions from another hospital. Nevertheless, in the medical context of Taiwan, people are likely to visit doctors between general hospitals due to physician’s reputation and affordable medical costs. The risk for subsequent self-harm in the patients from the two hospitals and the source of patients that nurses care for in these two hospitals are regarded as similar due to the approximate characteristics (e.g. mean age and gender ratio) of the patient source to that of a case register study in Taipei [40]. Thus recruitment from the two medical settings would not raise serious problem in interpretation of results. Secondly, the outcome of scale prediction was repeated self-harm. The results derived from such measurement may not be comparable to that of completed suicide. But repetition of self-harm was recognized as an important risk factor more prevalent than suicide ideation and has been adopted in other studies [22,37]. Therefore we regarded this to be an acceptable outcome measurement. Thirdly, we failed to recruit more patients and/or follow up the participants for longer than 6 months due to resource shortage, thus generalization was limited to emergency attendees with self-harm and the exclusion of those with severe physical complications after the index self-harm acts, cognitive dysfunctions, or those who refused to participate at baseline assessment. Given the fact that we failed to collect 6-month data in the control group which may affect the richness of the results and limit the analysis of this group, we focused on establishing the psychometric properties using the case group in this study and had the follow-up period longer than that of other study, e.g. three months [37]. To have a better understanding of patient source of the study groups and the limitation of the hospital controls in this study, refer to our previous publication [41]. Finally, there were factors not considered to be tested in the study but might regarded as repetition risk predictors, such as impulsivity, drug abuse, and life event. However, some of those factors were unforeseen (e.g. life event) and not detrimental to predict 6-month repetition of self-harm [37], so the influence of not including those in the study would be small. Future studies could validate using the CSPS in predicting completed suicide in longitudinal studies or generalizing the results to longer term risk prediction for higher risk of repeated self-harm in a wider clinical populations.

## Conclusion

We concluded that the CSPS is a valid and brief instrument suitable for self-harm repetition prediction. It was a considerable instrument for suicide risk screening based on its brevity and feasibility. However, cautions should be paid to level of therapeutic relationships during assessment, staff workload and adequate training for wider clinical applications.

## Appendix

Mr. Wang is a 47 year-old man who admitted to the Metabolic Ward, day five after his debilitating condition with poor sugar control. He was mostly alone at bedside without a caregiver. His primary nurse found several parallel scars on his left arm during physical check-up. He just briefly explained that he got those scars from his work. According to electronic medical records, he had two prior admissions to the emergency room due to cutting wounds at left wrist with unknown causes. He had no other major diseases or service attendance records. The night shift nurse observed that he had poor sleep and almost kept awake for the night since admission. He disclosed his bad mood and poor concentration and also showed limited activity level or interests in watching television or reading. His appetite became poor since last few weeks, which caused his body weight to lose from 68 kg to 59 kg. He could take care of himself in basic activities of daily living, but he was lack of motivation to move or talk and appeared slow-motioned than ever before. He felt he was not himself like before, and these changes have seriously affected his life quality and work. While the primary nurse who concerned about his situation asked what had happened to him, he replied, “I have been feeling tightness on my chest for weeks, and difficult breathing too. I’m wondering why I have to stay alive and face all these problems? My family hates me because I drink, but that’s because I have so much tension from work that no one could imagine … I have suffered enough from my work. Even I worked hard, I still got sacked and then my wife and kid left me. Now I’m alone. I feel so sad but no one understands me, I just feel like ending my life…”

## Competing interest

The authors declare that they have no competing interests.

## Authors’ contributions

CY-W designed the study, wrote the first draft and revisions and searched for part of the funding; HC-H undertook part of the statistical analysis and collected quantitative data with CR-H; SI-W provided critical opinions in data interpretation; FJ-S offered practical support in statistics; SI-L contributed in the search of major funding, supervised clinical data collection, and critically revised the manuscript. All authors read and approved the final manuscript.

## Pre-publication history

The pre-publication history for this paper can be accessed here:

http://www.biomedcentral.com/1471-244X/14/44/prepub

## Supplementary Material

Additional file 1 The Chinese version of the SAD PERSONS Scale (CSPS). Click here for file
