# Supplementary material for: Validation of the Chinese SAD PERSONS Scale to predict repeated self-harm in emergency attendees in Taiwan
Source: BMC Psychiatry. 2014 Feb 17;14:44. doi: 10.1186/1471-244X-14-44 (PMC3942520; doi:10.1186/1471-244X-14-44)
Supplement: Additional file 1 — The Chinese version of the SAD PERSONS Scale (CSPS). [file 1471-244X-14-44-S1.doc]

**Additional file 1. The Chinese version of the SAD PERSONS Scale (CSPS)**

| **Item** | **Acronyms** | **Descriptions/ prompts (score)** | **General nurses’ opinions** |
| --- | --- | --- | --- |
| 1 | **S**ex | Male (1) |  |
| 2 | **A**ge | ≧45 or ≦19 (1) |  |
| 3 | **D**epression | Depressive disorders or past psychiatric service contact (2)/ Have you ever been diagnosed with depression or visited mental health services before? Are you hopeless? | *For people with serious physical illness such as cancer, be aware of depressive mood, hopelessness and prior contacts of mental health services. |
| 4 | **P**revious attempts | Past self-harm act(s) (1)/ Have you ever harmed yourself by using any method? | *Nurses perceived the need for training to inquire this. |
| 5 | **E**xcessive ethanol or drug use | Previous alcohol or other substance abuse (1)/ Have you ever used alcohol or medications/drugs over safe amount and caused functioning impairment? | *This can be screened with initial nursing assessment during admission. |
| 6 | **R**ational thinking loss | Organic brain syndromes or psychiatric symptoms (2)/ Do you hear or see things that you think others may not hear/see? |  |
| 7 | **S**eparated/wid-  owed/ divorced | Disadvantaged marital status or romantic relationships (1)/ Have you lost any cherished relationship recently or are you separated/ widowed/ divorced? | *Patients may not disclose their true marital status; its reliability relies on good therapeutic relationship. |
| 8 | **O**rganized self-harm act | Current self-harm being lethal or seriously harmful and requiring medical interventions (2) | *Nurses perceived the need for training to inquire this. |
| 9 | **N**o social support | Lack of support from family members or friends (1)/ Do you have significant others that can listen to you or provide support no matter what? | *Its reliability relies on good therapeutic relationship. |
| 10 | **S**tated future intent | Expression of future intent of self-harm repetition (2)/ Do you have any intent to harm yourself in the future? | *Nurses perceived the need for training to inquire this. |

* Items with specific points from the nurses.
